# CENP-W Plays a Role in Maintaining Bipolar Spindle Structure

**DOI:** 10.1371/journal.pone.0106464

**Published:** 2014-10-15

**Authors:** Agnieszka Kaczmarczyk, Kevin F. Sullivan

**Affiliations:** Centre for Chromosome Biology, School of Natural Sciences, National University of Ireland, Galway, Ireland; University of Virginia, United States of America

## Abstract

The CENP-W/T complex was previously reported to be required for mitosis. HeLa cells depleted of CENP-W displayed profound mitotic defects, with mitotic timing delay, disorganized prometaphases and multipolar spindles as major phenotypic consequences. In this study, we examined the process of multipolar spindle formation induced by CENP-W depletion. Depletion of CENP-W in HeLa cells labeled with histone H2B and tubulin fluorescent proteins induced rapid fragmentation of originally bipolar spindles in a high proportion of cells. CENP-W depletion was associated with depletion of Hec1 at kinetochores. The possibility of promiscuous centrosomal duplication was ruled out by immunofluorescent examination of centrioles. However, centrioles were frequently observed to be abnormally split. In addition, a large proportion of the supernumerary poles lacked centrioles, but were positively stained with different centrosomal markers. These observations suggested that perturbation in spindle force distribution caused by defective kinetochores could contribute to a mechanical mechanism for spindle pole disruption. ‘Spindle free’ nocodazole arrested cells did not exhibit pole fragmentation after CENP-W depletion, showing that pole fragmentation is microtubule dependent. Inhibition of centrosome separation by monastrol reduced the incidence of spindle pole fragmentation, indicating that Eg5 plays a role in spindle pole disruption. Surprisingly, CENP-W depletion rescued the monopolar spindle phenotype of monastrol treatment, with an increased frequency of bipolar spindles observed after CENP-W RNAi. We overexpressed the microtubule cross-linking protein TPX2 to create spindle poles stabilized by the microtubule cross-linking activity of TPX2. Spindle pole fragmentation was suppressed in a TPX2-dependent fashion. We propose that CENP-W, by influencing proper kinetochore assembly, particularly microtubule docking sites, can confer spindle pole resistance to traction forces exerted by motor proteins during chromosome congression. Taken together, our findings are consistent with a model in which centrosome integrity is controlled by the pathways regulating kinetochore-microtubule attachment stability.

## Introduction

Establishment of a bipolar spindle in mitotic cells is a critical aspect of maintaining genomic integrity [1], [2]. Two critical structures, the spindle poles and the kinetochores of the chromosomes, regulate the structure and function of the spindle through dominant effects on microtubule stability and organization [3]. The spindle pole is a particularly complex structure, incorporating both the nucleating center of the centrosome, the complex pericentrosomal matrix as well as microtubule motor related self-focusing activities [4], [5]. In addition to linkages between poles and kinetochores, linkage of astral microtubules to the cell cortex and to non-kinetochore microtubules of the spindle creates a complex and dynamic distribution of forces within the mitotic cell [6]. Maintenance of spindle pole integrity is essential as defects in spindle pole organization lead to multipolar mitoses and mitotic failure [7]. In particular, aberrations in centrosome number are frequent in tumor cells and contribute to mechanisms of chromosome instability in cancerous cells [8], [9]. Knowledge of the mechanisms of maintenance of spindle poles is thus of critical importance for understanding tumor cells and their adaptations and weaknesses.

Centrosomes, as the primary microtubule organizing centres in animal cells (MTOC), are the core of the spindle poles and therefore their numbers need to be strongly controlled, duplicating exactly once per cell cycle, around the time of the DNA replication, and not exceeding two at the entry to mitosis [5], [10]. During late G2, centrosomes separate in a two-step process and this is thought to generate the first cue for the formation of a bipolar spindle. The first step involves dissembling of the fibrous linker connecting the two centriole pairs, and the second involves the concerted action of microtubule-based motor proteins Eg5 kinesin and cytoplasmic dynein, which function to separate the two centrosomes, nucleating bipolar spindle formation [11], [12]. However, centrosomes are not required for bipolarity, as observed in plants and mammalian oocytes as well as experimentally induced acentrosomal cells which can form bipolar spindles through centrosome-independent pathways [6], [13], [14], [15]. Indeed, experiments in *Xenopus* egg extracts lacking both centrosomes or kinetochores demonstrated the self-organizing properties of microtubules which can establish a bipolar structure based on interactions with microtubule motors and cross-linking proteins alone [16].

During mitosis, the bipolar spindle is a highly dynamic structure organized through the concerted action of different processes occurring in the spindle, including microtubule dynamic instability, poleward microtubule flux, kinetochore-directed chromosome movements, motor-driven antiparallel microtubule sliding, dynein-dynactin dependent minus-end directed microtubule transport and orientation movement of the spindle poles through cortical attachments [6]. Within the mitotic spindle, microtubule dynamics seem to be regulated mainly by microtubule-associated proteins (MAPs), that are required for focusing of the minus-end of the microtubules to form spindle poles [17], [18]. Principal MAPs represent nuclear protein of the mitotic apparatus (NuMa) and targeting protein for Xklp2 (TPX2), that bind and stabilize microtubules. Conversely, the plus-ends of microtubules are embedded at kinetochores, where various of microtubule plus-end associated proteins control the polymerization state of the kinetochore microtubules [3]. These include proteins promoting microtubule polymerization, such as MAP215, EB1 or CLASP family proteins; as well as microtubule depolymerases, such as kinesin-13 family proteins (MCAK) and kinesin-8 family members [19], [20]. In addition, it has been shown, that in mammalian cells the kinetochore activity itself influences mechanisms of spindle pole organization, by TPX2 microtubule cross-linking activity, which was found to be sufficient to maintain focused poles even in cells deficient in Nuf2, NuMa and HSET activities [21].

The kinetochores are assembled during G2 phase of the cell cycle at the centromere and these proteinaceous structures serve as the attachment point for microtubules on each sister chromatid [3]. The core of the centromere chromatin consists of the histone H3 variant containing CENP-A nucleosomes and the constitutive centromere-associated network (CCAN), a group of over 15 tightly associated chromatin proteins that forms the foundation on which the kinetochore and spindle assembly checkpoint components (SAC) assemble [22]. They coordinate the binding of a number of microtubule motor proteins, such as kinesin-related proteins CENP-E and MCAK as well as cytoplasmic dynein; and integrate this binding with the continuous poleward flux of tubulin subunits within the spindle lattice [3], [4], [23]. The CCAN also provides direct linkage between centromeric chromatin and the core microtubule binding Ndc80 and the KMN complex, through two distinct pathways. One is mediated through CENP-C's interaction with the Mis12 complex [24], [25] while CENP-T binds directly to the Spc24, 25 subunits of the Ndc80 complex [26]. The CCAN thus coordinates a number of microtubule binding and regulatory activities at the surface of the chromosome.

The CENP-T/W/S/X complex appears to assemble as a nucleosome-like tetramer [27] or octamer [28] to chromatin of the centromere. While CENP-T functions as a chromatin-linked receptor for the Ndc80 complex [26], CENP-S and –X also function at sites of DNA damage in recruitment of FANCM [29]. The complex assembles at centromeres during late S and G2 phases of the cell cycle and this assembly is essential for mitosis in each cell cycle [30], [31]. The CENP-S and –X components are dispensible in chicken DT40 cells for mitosis, while CENP-T and –W are both required. In human cells, we found that depletion of CENP-W induced a more rapid and profound disruption of mitosis than depletion of CENP-T and that this was associated with a pronounced multipolar spindle phenotype. Here, we investigate the mechanism of mitotic spindle pole defects induced by loss of CENP-W and find that CENP-W is required to maintain spindle pole integrity after establishment of bipolar spindles in HeLa cells. Our results suggest that CENP-W may play a regulatory role in kinetochore-microtubule binding, consistent with a role for the CCAN in regulating kinetochore microtubule function.

## Materials and Methods

### Cell culture, RNAi and drug treatment

HeLa CCL-2 cells were purchased from ATCC (American Type Culture Collection) and were grown in Dulbecco's Modified Eagle Medium (DMEM) with 4.5 g/L glucose (Lonza Biologics,Slough, United Kingdom) stable L-glutamine, 10% fetal calf serum (FCS), non essential amino acids (NEAA) and 1% penicillin streptomycin (PS). Culturing of cells was carried out in a Class III Bio-safety cabinet. Cells were maintained in 5% CO_2_ at 37°C. For cells stably expressing either GFP-CENP-W (a gift from Dr. Cheeseman) or mCherry-α-tubulin, a medium with selective antibiotics (blasticidin or G418, respectively) was used.

In siRNA experiments, synthetic double stranded ON-TARGET plus SMARTpool sequences (Dharmacon) for CENP-W (L-032901-01), CENP-T (L-014577-01) and non-targeting control pool (D-001810-10) were used. Targeted duplexes were transfected at 100 nM concentration into cells using DharmaFECT 1 reagent (Dharmacon), according to the manufacturer's instruction. Cells were analyzed 24 h and/or 48 h post-transfection.

To inhibit microtubule dynamics cells were incubated with nocodazole 1 µM. To analyze spindle pole fragmentation, cells were incubated with 100 µM monastrol (all from Sigma). All of the treatments were performed for the last 4 h before harvesting.

### Immunofluorescence

Cells were fixed in either 4% PFA for 20 min, in ice-cold methanol for 10 min (for γ-tubulin and centrin antibodies) or for 10 min in 1× PTEMF extraction buffer (20 mM PIPES, pH 6.8, 10 mM EGTA, 1 mM MgCl 2, 0.2% Triton X-100 and 4% PFA) prepared fresh from 4× stock solution (for all the mitotic checkpoint antibodies). Cells were permeabilized by 2×3 min wash in 0.1% Triton-X-PBS (PBS-TX), followed by blocking in PBS-TX containing 1% BSA for 30 min at room temperature, before being processed for immunofluorescence. Primary antibodies were diluted in 1% BSA in 1× PBS-TX in a 37°C incubator for 1 hr. Primary antibody dilutions were as follows: mouse anti-α-tubulin (DM1α, Sigma, 1∶1000), rabbit anti-γ-tubulin (Sigma, 1∶1000), rabbit anti-pericentrin (Bethyl, 1∶300), mouse anti-centrin-3 (Abnova, 1∶500), rabbit anti-TPX2 (Bethyl, 1∶100), mouse anti-Hec1 (GeneTex, 1∶100), human anti-centromere antibody (ACA, 1∶2000), mouse anti-BubR1 (Millipore, 1∶50), mouse anti-Mad1 (a kind gift from Dr. Andrea Musacchio, 1∶300), sheep anti-Mad2 (a kind gift from Dr. Steven Taylor's Laboratory, 1∶200), rabbit anti-Zwint-1 (Bethyl, 1∶100). Secondary antibodies conjugated to FITC, TRITC or Cy-5 (all from Jackson Laboratories) were chosen as appropriate and used as recommended by the supplier. DNA was counterstained with DAPI.

### Time-lapse live cell microscopy

A HeLa cell line stably co-expressing a histone H2B-GFP and mCherry-α-tubulin, obtained as previously described [32], was used to visualize chromosomes and mitotic spindles. Cells were transfected with siRNA for 48 h before imaging. For studies of mitotic timing and/or defects, time-lapse images were acquired at 3 min intervals for 8–12 h using 60× oil objective. Images were captured using a DeltaVision Core system (Applied Precision) controlling an interline charge-coupled device camera (Coolsnap HQ2; Roper) mounted on an inverted microscope (IX-71; Olympus). Collected sequences were analyzed using ImagePro Plus 6.2 software to identify the time of mitotic events in individual cells. Data were collated for analysis in Microsoft Excel.

### Image analysis

Microscopy of fixed specimens was carried out using the Deltavision Core at 2×2 binning using a 100× oil objective at 0.2 µm z-sections. Immunofluorescence images were subject to iterative constrained deconvolution and maximum intensity projection using the SoftWoRx software (Applied Precision).

Following immunofluorescence and data collection, deconvolved and projected images were analyzed in Image Pro Plus software. Using its analysis tool, kinetochores were defined by threshold segmentation Zwint-1 immunofluorescence or GFP-CENP-W. Next, kinetochore masks were applied from reference channel into the experimental channel (Hec1, BubR1, Mad1 and Mad2) staining and intensity values for each object were recorded. Finally, the collected fluorescence intensity values of both reference and experimental channel were background corrected for quantitative analysis of Hec1, BubR1, Mad1 and Mad2 abundance at the kinetochores. These analyses and statistical summary analysis was performed in Microsoft Excel.

### Western blotting

Whole cell extracts equivalent to 100,000 cells were separated by SDS-PAGE, transferred to PVDF membranes (Millipore), and processed for immunodetection. The membranes were incubated with primary antibodies: mouse anti-GFP (Roche, 1∶1000), mouse anti-tubulin (DM1α, Sigma, 1∶5000), rabbit anti-CENP-W (Abcam, 1∶1000) and rabbit anti-TPX2 (Bethyl, 1∶1000). Goat anti-mouse or goat anti-rabbit HRP antibodies (Jackson Laboratories) were successively applied. Antigens on the membrane were revealed by enhanced chemiluminescence (ECL, Milipore). Densitometric analysis of immunoblots were determined using the Gene Snap software from Syngene.

### DNA plasmid preparation and transfection

Plasmid expressing mCherry-**α**-tubulin was constructed by PCR amplification of **α**-tubulin 1b (TUBA1B) from HeLa cDNA followed by cloning into a Gateway adapted pEGFP-C1 derivative containing mCherry as the fusion protein partner. Mini plasmid DNA preparations of photoactivatable (PA)-GFP and pYFPC3-TPX2 (a kind gift from Dr. Isabelle Vernos) constructs were generated using Qiagen Mini Prep kit following the manufacturer's instructions. Transfection of GFP-CENP-W HeLa cells with plasmid DNAs was carried out by electroporation using a NucleofectorTM II device (Lonza Biologics,Slough, United Kingdom). Briefly, HeLa cells were trypsinised and 1×10^6^ cells for each DNA plasmid were spun in a centrifuge at 1200 r.p.m for 5 min. The supernatant was removed from cell pellet, 4 µg of plasmid DNA was pipetted directly on top of the cell pellet followed by 100 µl of Ingenio electroporation solution (Mirus supplied through Cambridge Bioscience, Cambridge, United Kingdom). The cell pellet was resuspended in this solution and transferred to an Ingenio cuvette (Cambridge Bioscience, Cambridge United Kingdom). The Amaxa program I-013 was selected and cells were transfected. Following nucleofection, cells were placed on a glass coverslips in 12-well dishes with pre-warmed medium and placed back in 37°C incubator, allowing the cells to grow and adhere before siRNA transfection. The siRNA administration was carried out 24 h later and transiently transfected cells were analyzed 24 h or 48 h later by fluorescence microscopy or Western blotting.

## Results

### Spindle dynamics in CENP-W depleted cells

**Figure 1:**
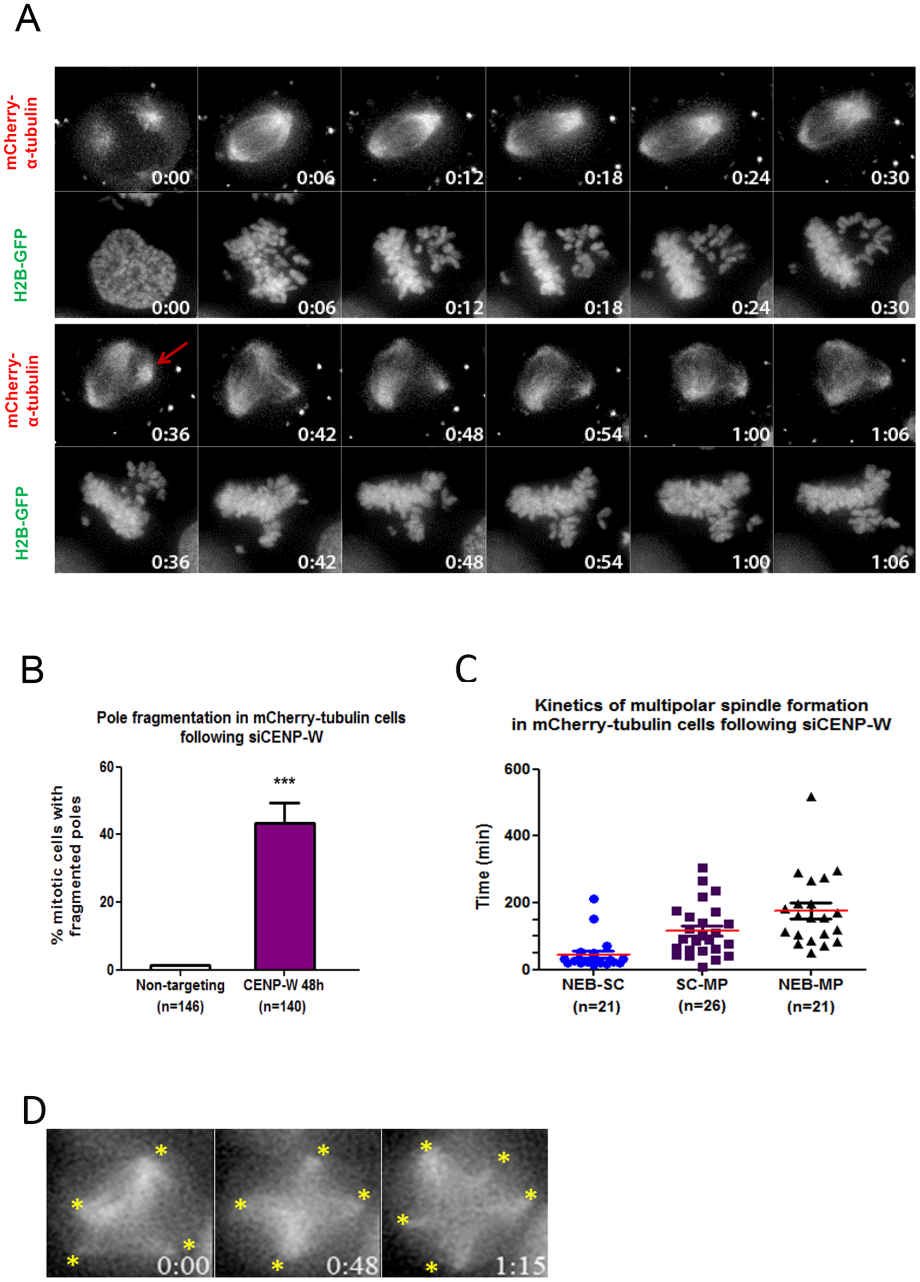
Tubulin labeling confirms spindle pole fragmentation in CENP-W depleted cells. (**A**) HeLa H2B-GFP cells were transfected with mCherry-α-tubulin to directly visualize spindle MTs following depletion with pooled siCENP-W for 48 h. It is clear that the cell initially possessed a bipolar spindle (time 0–30 min) but then a supernumerary pole appeared under mCherry-tubulin fluorescence (36, red arrow). Spindle poles moved apart and the chromatin re-organized within the multipolar spindle. Time = hr:min. (**B**) Graph represents mean percentage (+SEM) of the pole fragmentation events occurring in mitotic cells following CENP-W depletion. *** p<0.0001 (one-way ANOVA followed by Dunnetts multiple comparison). (**C**) Graph represents quantitative measurements (+SEM) of mitotic kinetics in CENP-W depleted cells: timing from nuclear envelope breakdown to chromosome scattering (NEB-SC), from scattering to multipolarity (SC-MP) and cumulative result from nuclear envelope breakdown to multipolarity (NEB-MP) (**D**) Selected images from time-lapse imaging of cells expressing mCherry-α-tubulin following CENP-W depletion. The yellow stars indicate the spindle poles. Time = hr:min.

Investigation of the consequences of CENP-W depletion in HeLa cells revealed a high frequency of multipolar spindle formation, suggesting difficulties in either the formation or maintenance of bipolar spindles [30]. This could be due to cells entering mitosis with more than two centrosomes to form multipolar spindles directly, or to defective behavior of spindle poles during mitosis. To address this, HeLa cells expressing mCherry-tubulin and GFP-histone H2B were depleted of CENP-W and observed by live cell fluorescence microscopy. These observations revealed that the majority of cells form a bipolar spindle on entry into mitosis, followed by subsequent fragmentation of the spindle poles associated with large scale rolling of spindles within the cells (**Figure 1A**). In this example a mitotic cell exhibited a bipolar spindle at the beginning of the observation (0–30 min). While the chromosomes initially dispersed within a bipolar spindle, a new supernumerary spindle pole appeared (36 min), and the spindle re-organized to a tri-polar structure with chromosomes adopting an appropriate configuration. Quantitative analysis of observed mitoses (n = 140) demonstrated that over 40% of mitotic cells within the CENP-W depleted population increased the number of their poles, going from bipolar to tri-, tetra- and multipolar cells within the duration of the 12 h imaging (**Figure 1B and D**). This contrasts with 1.3% spontaneously occurring pole fragmentation observed in control cells.

Extended mitotic arrest leads to cohesion fatigue and mitotic spindle abnormalities similar to those reported here [33], [34]. We compared the effects of CENP-W depletion with cohesion fatigue through kinetic analysis of chromosome scattering behavior and spindle multipolarization. CENP-W depleted cells exhibited chromosome scattering quickly, with 76% of mitoses affected within 44 minutes of nuclear envelope breakdown (NEBD (**Figure 1C**)). This was also rapidly followed by spindle pole fragmentation, which occurred within the first 2 h upon NEBD in ∼40% of siCENP-W-treated mitotic cells. These events are substantially more rapid in onset than those attributed directly to cohesion fatigue in HeLa cell models [33], [34]. Further, immunostaining for Zwint-1, a marker of the outer kinetochore, showed intact chromosome pairing in a preponderance of chromosomes scattered at poles (**Figure S1A in File S1**). Unpaired kinetochores are observed in CENP-W depleted cells, indicating that cohesion fatigue does occur, but this is most likely in cells arrested for lengthy periods in mitosis. While cohesion fatigue may play a role in the long-term outcome of CENP-W depletion, the rapid kinetics of chromosome scattering and spindle multipolarity suggested a more direct link between a kinetochore defect and spindle pole fragmentation in CENP-W depleted cells.

### Centrosomal abnormalities following CENP-W depletion

The observation that pole fragmentation events occur from originally bipolar spindles indicates that centrosome/spindle pole complexes are compromised in their integrity as a consequence of CENP-W depletion. However, since most of the pole fragmentation events were observed 48 h following transfection with siRNA, we could not rule out the possibility of amplified centrosomes from the first cell cycle being ‘carried over’ to the next cell cycle and therefore giving rise to clustered multipolar spindles in the second mitosis following CENP-W depletion. Alternatively, the pole fragmentation could arise from premature centriole disengagement [35], [36] or due to fragmentation of pericentriolar material following CENP-W depletion.

**Figure 2:**
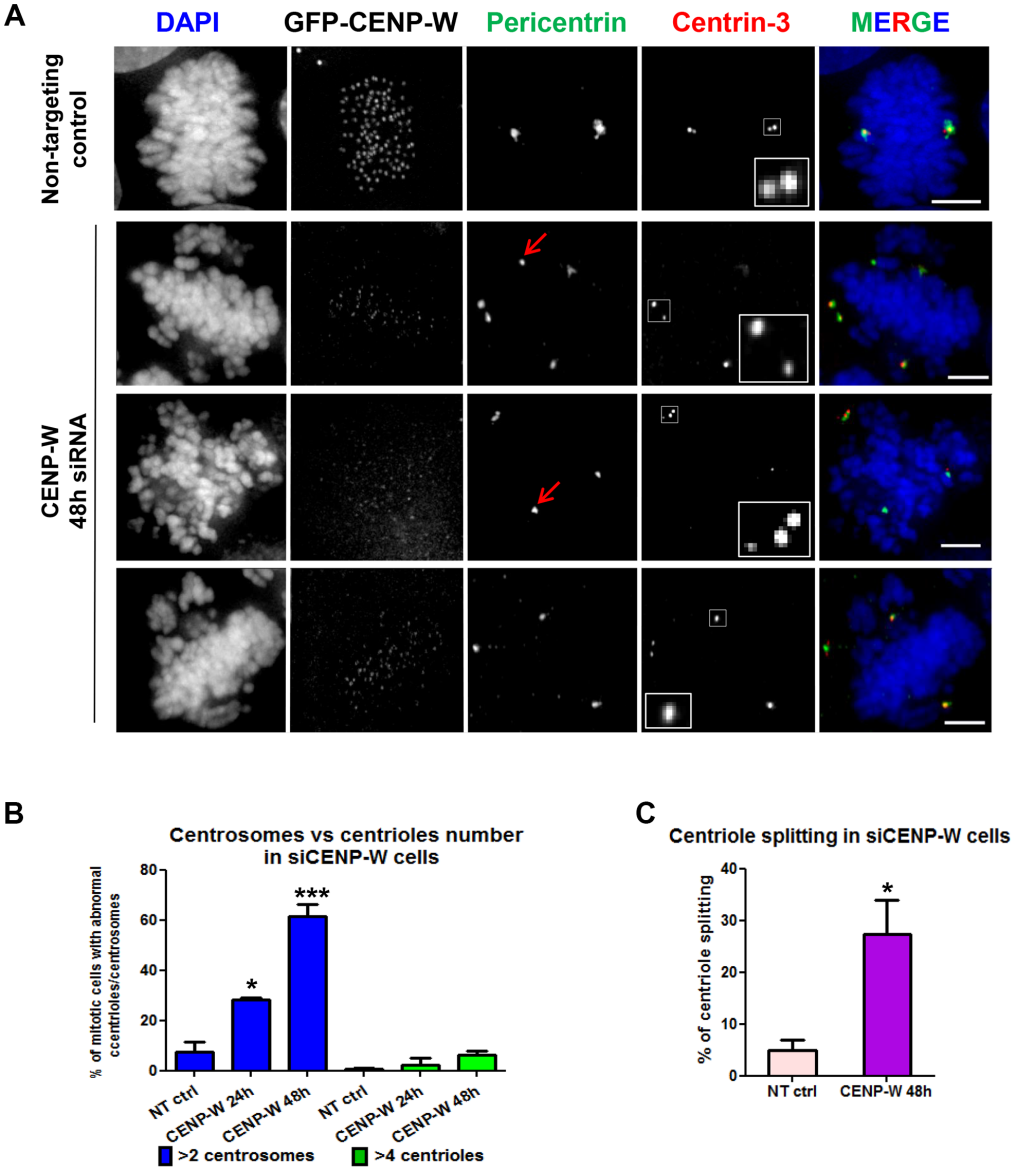
Pole fragmentation is correlated with abnormal centriole splitting in CENP-W depleted cells. (**A**) Representative panel of GFP-CENP-W cells transfected with siCENP-W for 48 h, stained for pericentrin (for centrosome detection, green) and centrin-3 (for centriole detection, red). siCENP-W prometaphase cells exhibited multipolar spindles (pericentrin panel, red arrows indicate non-centriolar associated poles). (**B**) Graph represents mean percentage (+SEM) of mitotic cells with >2 pericentrin or >4 centrin foci (at least 100 cells were scored, in triplicate assays). (**C**) Graph represents mean percentage (+SEM) of mitotic cells with split centrioles. * p<0.01, *** p<0.0001 (one-way ANOVA followed by Dunnetts multiple comparison). Scale bar 5 µm.

To evaluate the possibility of promiscuous centrosome duplication following CENP-W depletion, we examined centriole number in HeLa cells stably expressing GFP-tagged CENP-W, allowing us to correlate centriole number with the extent of depletion in individual cells (**Figure S2 in File S1**). Using a 48 h siRNA transfection protocol both tagged and endogenous CENP-W were reduced to approximately 20% of control levels by Western blot (18% and 22% respectively; **Figure S2A in File S1**) while the CENP-W-GFP reporter showed comparable levels of knockdown at mitotic centromeres by fluorescence microscopy (**Figure S2B in File S1**). The number and distribution of centrioles was determined by staining cells for centrin-3 (**Figure 2A**), revealing that 95% of cells contained a normal complement of 4 centrioles following CENP-W depletion despite abnormal numbers of centrosomal foci, assayed as pericentrin (**Figure 2B**). Abnormal splitting of centriole pairs was observed in approximately 30% of mitotic cells, defined as centrioles positioned alone or with greater than about one diameter separation (**Figure 2A and 2C**). Consistent with live cell studies, a major increase of supernumerary foci containing these components was observed following CENP-W depletion, indicating that the fragmented spindle poles observed in live cell studies are comprised of fragmented centrosomal material. These often lacked any associated centriole (red arrows in **Figure 2A** **and Figure S1B in File S1**). The increase in supernumerary poles was observed rapidly, with almost 30% of cells showing supernumerary centrosomal foci 24 h post-transfection, (**Figure 2B**). Taken together with live cell studies, these findings demonstrate that spindle multipolarity in CENP-W depleted cells do not originate from centriolar or centrosome overduplication per se, but is generated during mitosis associated with fragmentation of centrosomal material and to a lesser extent with abnormal centriole splitting.

### Kinetochore associated molecular dysfunction of CENP-W depleted cells

**Figure 3:**
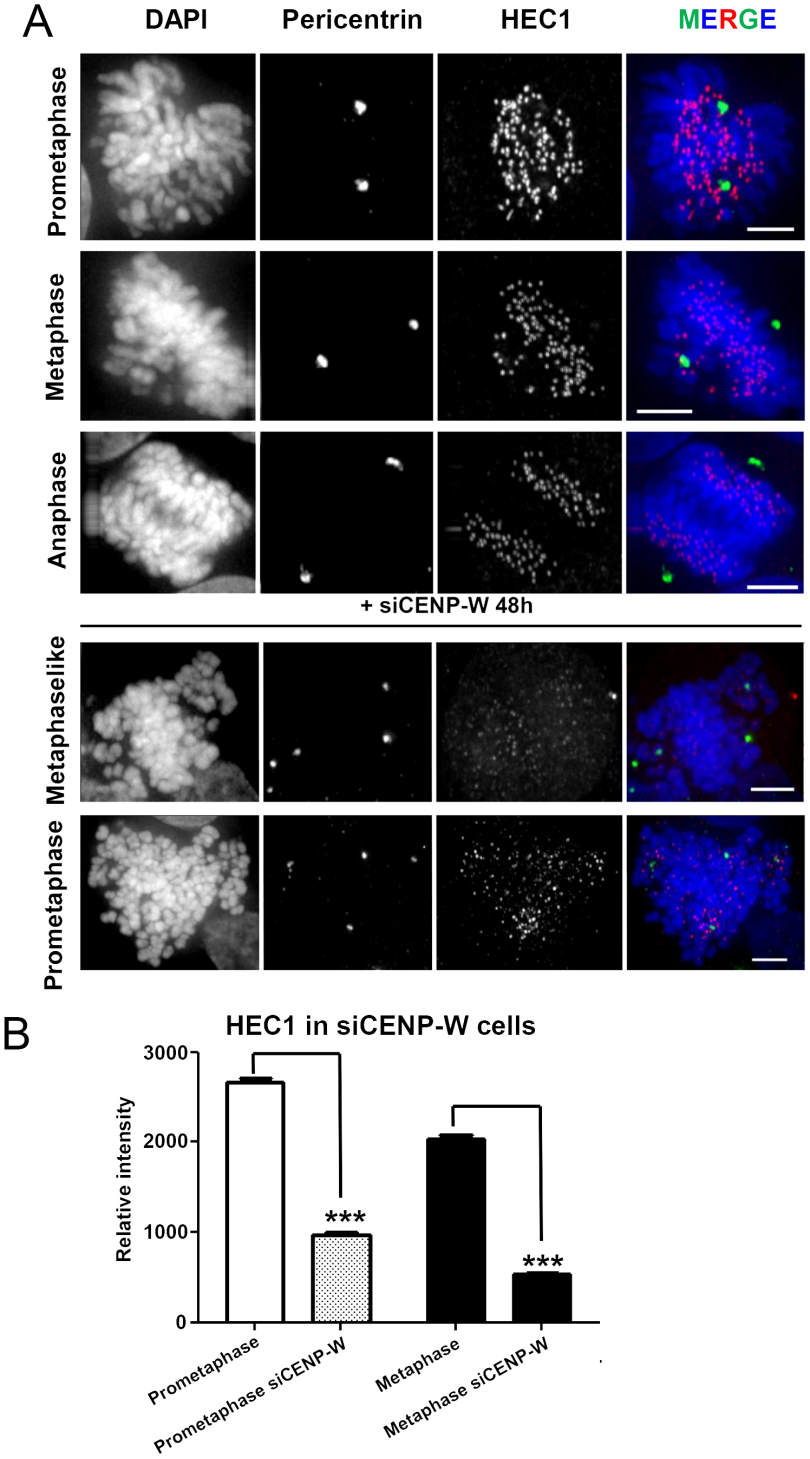
Impaired kinetochore binding of Hec1 protein following CENP-W depletion. (**A**) Representative panel of GFP-CENP-W cells transfected with siCENP-W for 48 h, and stained for pericentrin (green) and Hec1 foci (red) across different mitotic stages, scale bar 5 µm. (**B**) Graph representing quantitative measurements of the mean (+SEM) of relative Hec1 fluorescence signal intensity of the mitotic prometaphase and metaphase cells, following siRNA transfection. Values plotted were background corrected (at least 1000 kinetochores were scored in duplicate assays). *** p<0.0001 (one-way ANOVA followed by Dunnetts multiple comparison).

No direct interactions have been reported between CENP-W and components of the centrosome nor has CENP-W been shown to localize to centrosomes or spindle poles in human cells [37]. The CENP-T/W complex does, however, directly interact with components of the KMN complex, including the core microtubule binding protein Hec-1/Ndc80 [26] and Hec1-depleted cells display mitotic defects similar to CENP-W depleted cells, with prometaphase-like mitotic arrest and disorganized spindles [38], [39]. Examination of kinetochore associated Hec1 by immunofluorescence demonstrated that CENP-W depletion resulted in a significant reduction of Hec1 with some residual occupancy of the protein during the earlier stages of mitosis (**Figure 3A and B**). We investigated further defects of CENP-W depleted kinetochores and examined their ability to support mitotic checkpoint signaling, by staining for different SAC proteins. CENP-W depleted mitotic HeLa cells accumulated high levels of BubR1 (**Figure S3A in File S1**) but we found reduced binding of Mad1/Mad2 complex to the kinetochores (**Figure S3B, C in File S1**), consistent with previous findings showing the requirements of Hec1 for targeting Mad1/Mad2 checkpoint complex to the kinetochores [38]. The presence of BubR1 accounts for the robust mitotic arrest following CENP-W depletion and also illustrates the selectivity of the lesion caused by loss of CENP-W. Therefore, a selective defect in kinetochore assembly is the most likely primary consequence of CENP-W loss and the probable cause of multipolar spindle formation and centrosomal abnormalities reported above.

### CENP-W phenotype suppression following drug treatment

**Figure 4:**
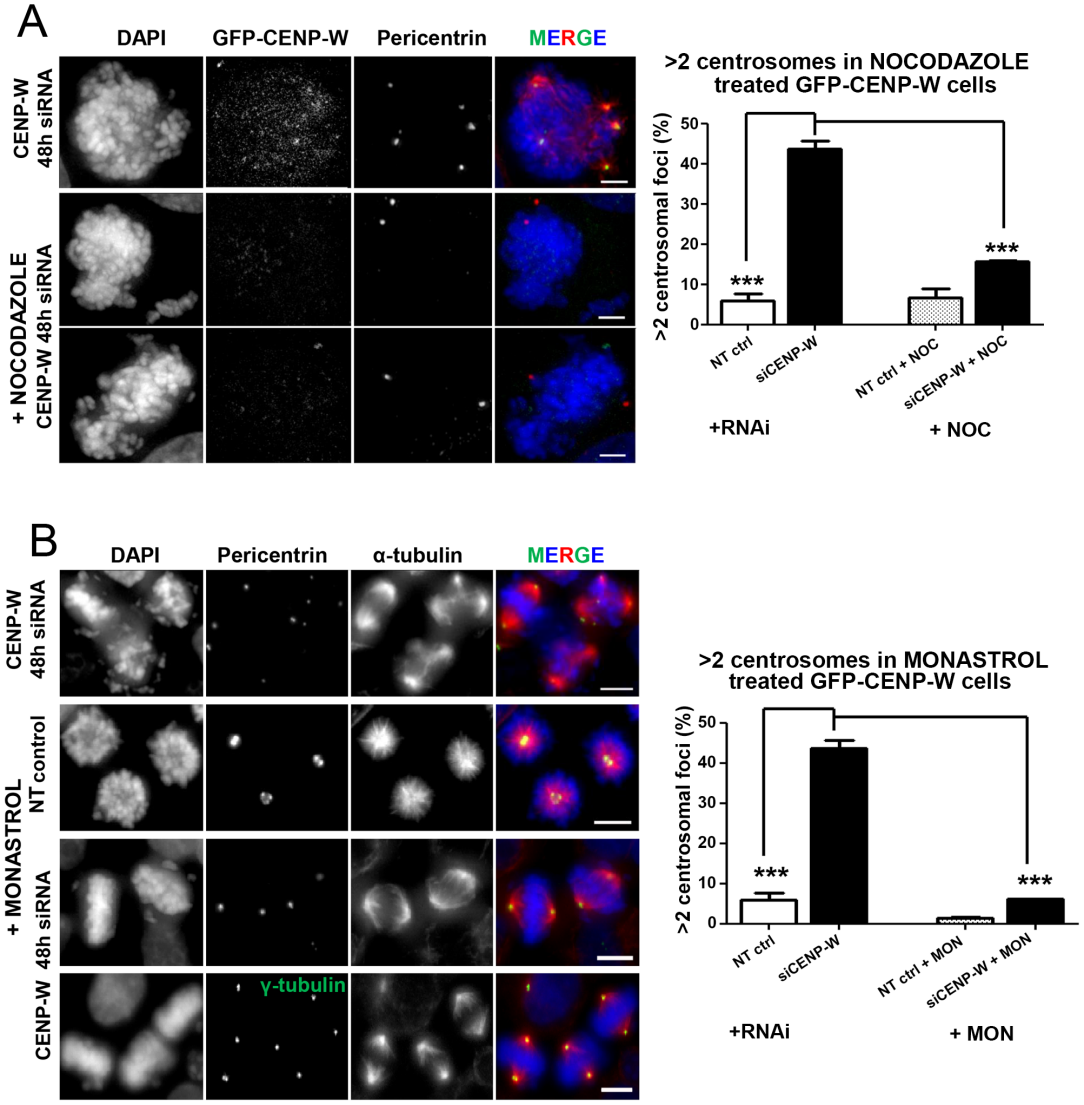
Suppression of CENP-W-dependent multipolarity following drug tratments. (**A**) Suppression of siCENP-W-dependent spindle pole fragmentation by nocodazole. Representative panel of GFP-CENP-W cells transfected with siCENP-W for 48 h, incubated with 1 µM nocodazole (NOC) for the last 4 h of transfection and stained for pericentrin (green) and α-tubulin (red). Graph represents mean percentage (+SEM) of the mitotic cells with supernumerary centrosomes (based on pericentrin and γ-tubulin foci) following nocodazole and/or siRNA treatment (at least 100 cells were scored in duplicate assays). (**B**) Monastrol treatment suppresses centrosome fragmentation in CENP-W depleted cells. Representative panel of GFP-CENP-W cells transfected with siCENP-W for 48 h, incubated with monastrol (MON) for the last 4 h of transfection and stained for pericentrin or γ-tubulin (green). Graph represents mean percentage (+SEM) of the mitotic cells with supernumerary centrosomes (based on pericentrin and γ-tubulin foci) following monastrol and/or siRNA treatment. At least 100 cells were scored, in duplicate assays. *** p<0.0001 (one-way ANOVA followed by Dunnetts multiple comparison). Scale bar 5 µm (A) or 10 µm (B).

Analysis of live cells depleted of CENP-W revealed that all observed instances of spindle pole fragmentation occurred after formation of initially bipolar spindles (**Figure 1A**). To examine the role of spindle forces in these processes, we treated CENP-W depleted cells with inhibitors of spindle function. To determine whether centrosome fragmentation was dependent on spindle microtubules, cells were treated with high dose nocodazole (1 µM) to induce nearly complete depolymerization of microtubules [40]. Examination of cells by immunofluorescence with centrosome markers (pericentrin and γ-tubulin) revealed intact centrosomes near the periphery of the cells and the frequency of aberrant centrosome numbers observed in CENP-W depleted cells was significantly reduced (**Figure 4A** **and Figure S4 in File S1**). We can conclude that spindle pole fragmentation is dependent on microtubule function in these cells.

**Figure 5:**
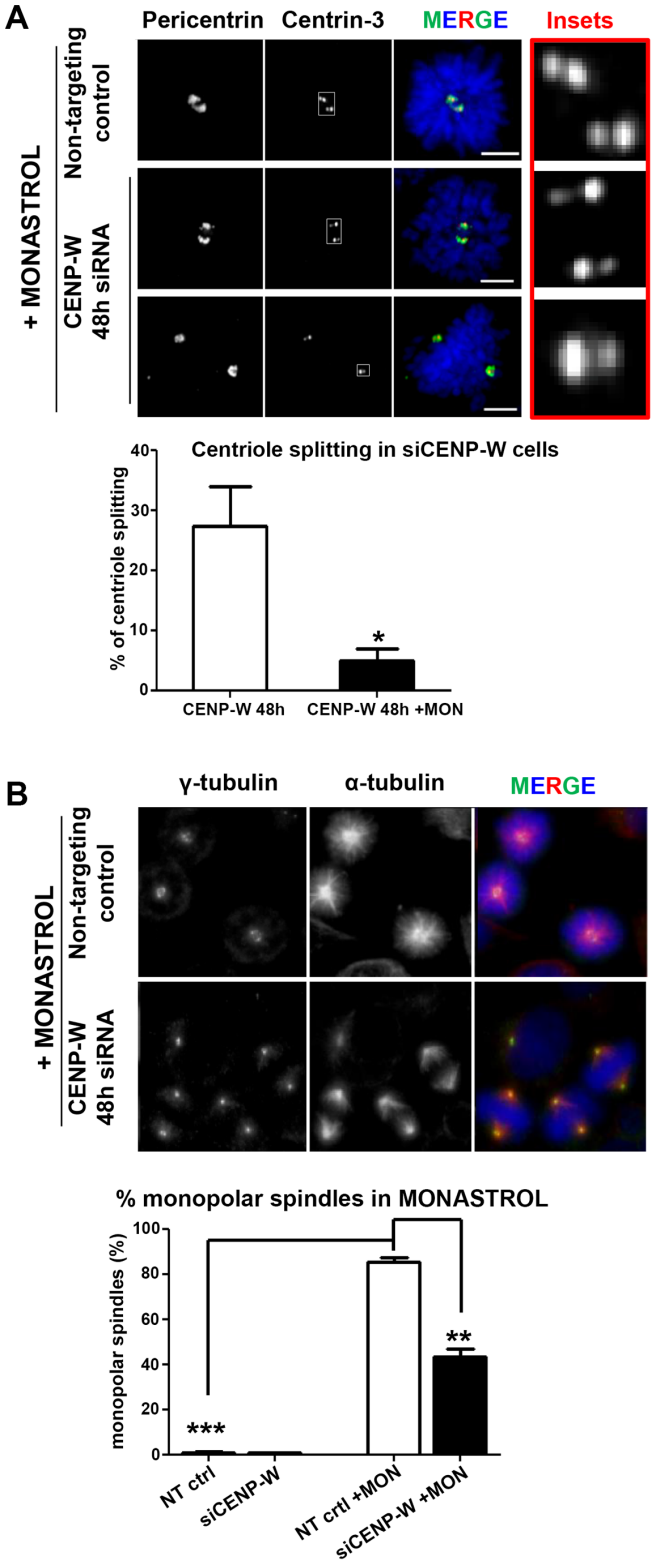
Monastrol treatment rescues abnormal centriole splitting. (**A**) Representative panel of GFP-CENP-W cells transfected with siCENP-W for 48 h, incubated with monastrol (MON) for the last 4 h of transfection and stained for centrin-3 (centriole, red). The boxed enlargements show centriole pairs in control and siCENP-W treated cells following monastrol incubation. Graph represents mean percentage (+SEM) of the mitotic cells with split centrioles following monastrol and/or siCENP-W treatment. (**B**) CENP-W depletion counteracts monopolarity in monastrol treated cells. Representative panel of GFP-CENP-W cells processed as described in (A) and stained for γ-tubulin (green) and α-tubulin (red). Graph represents mean percentage (+SEM) of the mitotic cells with monopolar spindles following monastrol and/or siRNA treatment. At least 100 cells were scored, in duplicate assays. *** p<0.0001 (one-way ANOVA followed by Dunnetts multiple comparison). Scale bar 5 µm (A) or 10 µm (B).

To examine whether a bipolar microtubule array is required for the observed spindle pole disruption in CENP-W depleted cells we applied monastrol (MON). This widely used drug acts at the entry into mitosis, preventing the separation of centrosomes through Eg5 kinesin inhibition and arresting the cells in mitosis with monopolar spindles [41]. HeLa GFP-CENP-W cells were depleted of CENP-W and exposed to a 4 h treatment with 100 **µ**M monastrol, 44 h post-transfection with siRNA. More than 90% of control MON-treated cells displayed two pairs of adjacent centrioles at the centre of the monopolar spindle (**Figure 4B**). CENP-W depletion prior to monastrol treatment resulted in three distinct features. Firstly, the appearance of supernumary centrosomal foci due to CENP-W depletion was suppressed. Quantitative analysis of centrosome foci using two centrosomal markers, pericentrin and γ-tubulin, revealed a significant decrease of supernumerary centrosomal foci in CENP-W depleted cells following monastrol treatment, comparable to the untreated control (**Figure 4B**). Secondly, monastrol treatment suppressed abnormal centriole splitting observed after CENP-W depletion (**Figure 5A**). A third, unexpected feature of the combined treatment, was the observation of bipolar spindles at high frequency in cells simultaneously depleted of CENP-W and treated with monastrol, with over 50% of observed mitotic spindles exhibiting apparently normal bipolar structure with relatively well organized chromosomes within metaphase plate and spindles (**Figure 5B**). Thus, CENP-W depletion and monastrol treatment reciprocally rescue their apparently independent effects on mitotic spindle structure.

Taken together the combined results of the two drug treatment experiments demonstrate that spindle pole fragmentation following CENP-W depletion is a microtubule-dependent process that requires Eg5 kinesin function. The data indicate that centrosome disruption and associated events of centriole splitting are most likely due to imbalance between kinetochore-dependent forces and Eg5-directed spindle effects that thus exert excessive shearing forces at centrosomes, tearing the latter apart and producing the supernumerary centrosomes observed.

### Overexpression of TPX2 rescues spindle pole fragmentation

A key protein that regulates integrity of the spindle pole is TPX2, a 98 kDa Ran-regulated, cross-linking microtubule-associated protein, that nucleates, binds and bundles microtubules *in vitro* [41]. It has been shown that its function is required for spindle pole organization, Aurora A activation and for microtubule formation at kinetochores [42], [43]. We hypothesized that overexpression of TPX2 could increase the mechanical stability and integrity of spindle poles through its microtubule cross-linking activity. To test this idea we expressed a fusion of yellow fluorescent protein and hTPX2 (YFP-TPX2) in HeLa GFP-CENP-W cells. As expected, YFP-TPX2 recombinant protein displayed a pattern of localization very similar to the endogenous protein as assayed by immunofluorescence (**Figure S5A and S6 in File S1**). Next, we evaluated the efficiency and persistence of the over-expressed fusion protein over time, by western blotting and fluorescent microscopy (**Figure S5 in File S1**). The tagged version of the protein remained detectable up to 60 h post-transfection, as depicted on the western blot and fluorescent images (**Figure S5B and C in File S1**). We did not observe any major mitotic defects following YFP-TPX2 overexpression, in contrast to a previous report using GFP-TPX2 [44]. Thus, exogenously expressed YFP-TPX2 is efficiently incorporated into mitotic spindles, presumably augmenting the endogenous protein.

**Figure 6:**
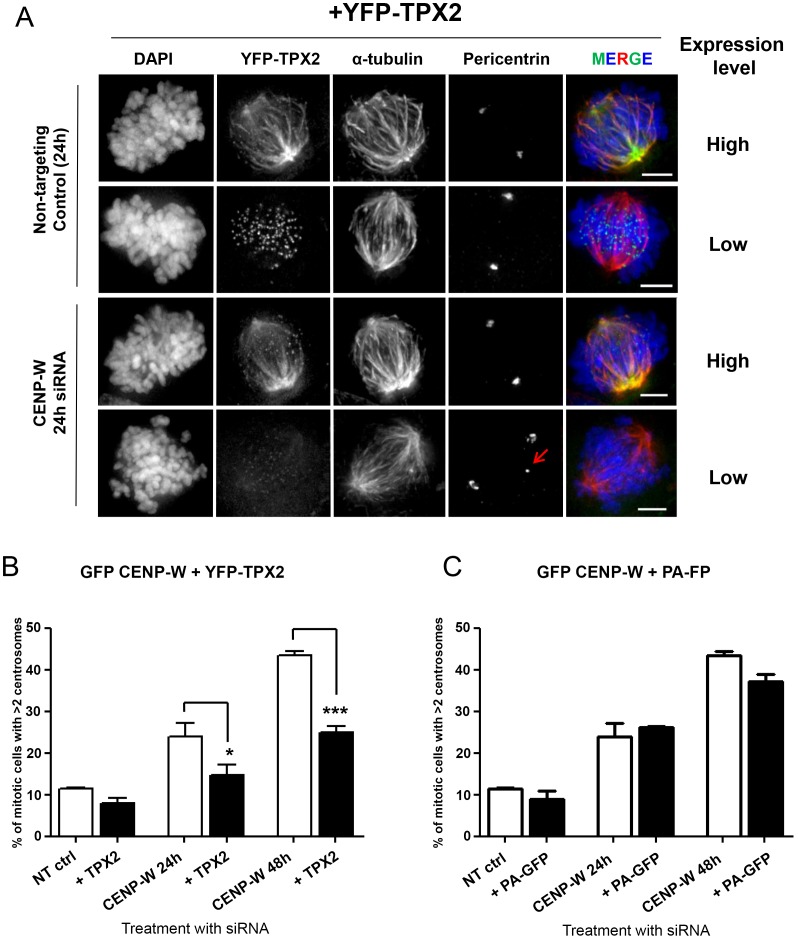
Overexpression of hTPX2 suppresses multipolarity in CENP-W depleted cells. (**A**) Representative panel of GFP-CENP-W cells transfected with YFP-TPX2 (green), followed by siRNA depletion of CENP-W and stained for α-tubulin (red) and pericentrin. In the overlay, yellow color indicates co-localization of red microtubules and green YFP-TPX2 overexpression. Red arrow indicates supernumerary pericentrin foci in siCENP-W cell with low levels of TPX2 overexpression. (**B–C**) Graphs represent mean percentage (+SEM) of the mitotic cells with supernumerary pericentrin foci following depletion of CENP-W in YFP-TPX2 or PA-GFP transfected cells (n = 150 mitotic cells, in triplicate assays). * p<0.01, ***p<0.0001 (one-way ANOVA followed by Dunnetts multiple comparison). Scale bar 5 µm.

To examine the functional consequence YFP-TPX2 overexpression under conditions of CENP-W depletion, HeLa GFP-CENP-W cells were transfected with YFP-TPX2 plasmid DNA then treated with siRNA 24 h later. Samples were fixed at 24 h and 48 h after siRNA treatment to assess the centrosomal organization in the first and the second cell cycles following CENP-W depletion. To control for the additional influence of plasmid transfection, cells were transfected in parallel with a plasmid expressing photactivatable green fluorescent protein (PA-GFP). Samples were processed for immunofluorescence with a pericentrin antibody to monitor centrosome integrity and formation of multipolar spindles. Cells were arbitrarily segregated into low and high expressors on the basis of YFP-TPX2 intensity (**Figure 6A**). Consistent with our hypothesis, cells displaying high levels of over-expressed YFP-TPX2 typically possessed two compact pericentrin foci following CENP-W depletion while low expressors showed a high frequency of multiple pericentrin foci in mitosis (see red arrow in **Figure 6A**), indicating the ongoing pole fragmentation in these cells. Quantitative analysis of pericentrin foci in mitotic cells revealed a substantive (35–40%) and significant decrease in supernumerary centrosomes, in both populations of a 24 h and 48 h post-transfection with siRNA (**Figure 6B**). Concurrent analysis of control PA-GFP transfected cells showed no significant difference in the appearance of the supernumerary pericentrin foci following CENP-W depletion in these cells (**Figure 6C**). We conclude that overexpression of TPX2 suppresses spindle multipolarity in CENP-W depleted cells.

## Discussion

The aim of this study was to investigate the mechanism of multipolar spindle formation following CENP-W depletion. As reported in previous studies, the CENP-W/T complex of the CCAN plays an essential role in mitosis [30], [37]. In our studies of depletion of CENP-T and –W, we found that CENP-W depletion gave a much more pronounced yield of multipolar spindles than CENP-T depletions done under the same conditions [30]. Depletion of CENP-W protein induces severe mitotic aberrations in human cells, namely defective chromosome congression leading to prolonged mitosis with disorganized prometaphase-like chromosomes scattering near the spindle poles and formation of multipolar spindles, associated with centriole splitting. Investigation of the spindle fragmentation phenotype of CENP-W depletion in HeLa cells reveals a defect compatible with aberrant function of the Ndc80 complex, which the CENP-T/W/S/X particle interacts with through CENP-T [26]. However, features of the multipolarity are indicative of a change in function rather than a simple loss of function of the Ndc80 complex, suggesting that CENP-W could play a role in modulating Ncd80 function, presumably through the heterotetrameric particle [27].

Multipolarity is a hallmark of tumor cells and may arise from supernumerary centrosomes in a cell as a consequence of centrosome overduplication or cytokinesis failure [36], [45], [46]. Loss of spindle pole integrity during mitosis is an alternative way leading to multipolarity independent of centrosome amplification, due to, for example, premature centriole disengagement or loss or alterations of centrosomal components [47], [48], [49], [50]. We distinguished these possibilities, ruling out centriole overduplication in CENP-W depleted cells by demonstrating that CENP-W depleted cells have normal centriole numbers despite an increase in centrosomal foci initiated during a short-term experiment. In fact, all spindle pole fragmentation we observed originated from apparently normal bipolar spindles, associated with some premature centriole splitting. Since CENP-W has not been reported to reside at spindle poles, we interpret these results as indicating that defective kinetochores depleted of CENP-W destabilize spindle structure by distorting force distribution in the spindle.

Alternatively, extended prometaphase delay produces spindle defects through the mechanism of cohesion fatigue [33], [34]. Time-dependent loss of cohesion leads to premature chromatid separation, centriole disengagement and pronounced spindle motion associated with spindle pole fragmentation. The phenotypes of CENP-W depleted cells are very similar in kind, however, but not in detail. Firstly, kinetic analysis of mitosis in HeLa cells depleted of CENP-W revealed that the initial spindle pole fragmentation events occurred almost immediately following bipolar spindle establishment, and at least several hours before centriole disengagement reported in HeLa cells exposed to prolonged metaphase arrest [34]. Moreover, following CENP-W depletion, only ∼25% of cells with supernumerary centrosomal foci demonstrated an abnormal centriole splitting, the multipolar phenotype most frequently associated with cohesion fatigue [33], [34]. The remaining 30–35% of cells containing supernumary spindle poles possessed acentriolar fragments in addition to spindle poles with normal paired centrioles. While some CENP-W depleted cells exhibit prematurely separated kinetochore pairs, it is likely that cohesion fatigue does arise in cells after extensive mitotic arrest due to CENP-W depletion. Nevertheless, the kinetics and mechanism of spindle pole fragmentation argue against a cohesion defect as a primary cause of multipolarity.

We observed a decrease in Hec1 occupancy at kinetochores as well as diminished Mad1/Mad2 abundance in CENP-W depleted cells. Hec1 depletion is not complete in our experiments and depletion of Hec1 [39] or Nuf2 [21] alone has not been reported to result in pronounced spindle multipolarity. Thus, loss of Hec1 binding alone does not explain spindle behavior in our experiments. However, expression of a dominant negative form of Hec1 (GFP-HEC1) has been shown to induce mitotic defects in HeLa cells with a high yield of multipolar spindles coupled with centriole splitting [51]. The Ndc80 complex interacts in multiple ways with microtubules, modulating microtubule dynamics as well as forming a dynamic binding element [52]. In budding yeast, CENP-T homologues bind to and modulate the function of Ndc80, suggesting that the complex can receive regulatory input from its centromeric chromatin binding partner [53], [54]. The features of CENP-W induced multipolarity discussed below suggest that the mammalian CENP-T/W/S/X particle could also modulate the function of the Ndc80 complex in addition to serving as a binding site.

While a dependence on microtubules is not surprising, the behavior of CENP-W depleted cells in the presence of monastrol was informative. By inhibiting antiparallel MT sliding with Eg5 inhibition, multipolar spindle formation was suppressed in CENP-W depleted cells, coordinately with rescue of centrosome fragmentation. In these cases, no evidence was observed for premature chromatid separation or centriole dissociation. Further, in the mitotic population of CENP-W depleted monastrol-treated cells we would expect to see cells with multipolar spindles that had been arrested during the first 44 h, before drug treatment. These were rarely observed. We concluded that multipolar spindles in CENP-W depleted cells require Eg5-mediated pushing forces for their formation and maintenance. Evidently, Eg5 is required for bipolar spindle formation only in the presence of normal kinetochore microtubule binding mechanisms, as also observed following dynein depletion [11]. Absent CENP-W, Eg5 participates in translation of shearing forces to spindle pole fragmentation. This could be through direct motor activity or through a cross linking function that transmits, for example, CENP-E directed forces, which have been shown to disrupt centrosome integrity following CLASP depletion [50].

The fact that TPX2 overexpression also suppresses multipolarity in CENP-W depleted cells also argues that the principle factor driving abnormal spindle structure is an imbalance in mechanical force distribution through the spindle. TPX2 exhibits many functions during the mitotic spindle assembly, such as binding and cross-linking microtubules and focusing spindle poles [18], [44]. Moreover, it has been found to influence spindle assembly and maintain focused spindle poles even in the absence of other stabilizing factors, such as NuMa or HSET [21]. In our experiments we did not observe a phenotype for YFP-TPX2 overexpression alone (Figure 6), in contrast to reports that GFP-TPX2 expression resulted in spindle abnormalities [44]. Western blot analysis suggests that endogenous TPX2 undergoes autoregulation under these experimental conditions (Figure S5 in File S1). The fact that its overexpression suppressed multipolar spindle formation in CENP-W depleted cells suggests that enhanced spindle pole integrity can overcome the imbalance in force distribution caused by the defective kinetochores.

While our TPX2 rescue experiment was modeled on the basis of the microtubule cross-linking activity of TPX2, an alternative explanation of the results is through direct biochemical action on Eg5. TPX2 binds to and regulates the activity of Eg5, directly reducing motor activity in microtubule gliding assays [55], [56]. Disruption of TPX2-Eg5 interaction induced spindle disorganization and pole fragmentation as well as disrupted Eg5 distribution on the spindle [55]. Eg5 is reciprocally required for proper TPX2 distribution on the spindle and both require dynein function as well [42], [57]. In this model, overexpressed TPX2 works similarly to monastrol in inhibition of Eg5-dependent force generation, acting to directly suppress microtubule-dependent shear forces rather than protect against them. In either case, a dysregulated Eg5 is playing a role in disrupting spindle structure in CENP-W depleted cells, indicating that proper CCAN function is required for coordinated regulation of spindle architecture. Thus, while we cannot distinguish between a direct pole stabilizing mechanism versus an Eg5-dependent mechanism to explain the TPX2 rescue effect, these experiments support a spindle-directed mechanism for CENP-W-dependent multipolar spindle formation as opposed to cohesion fatigue.

To our knowledge, no other CCAN subunit possesses such a strong potency to affect spindle polarity as CENP-W, with the exception of CENP-L and CENP-O, which depletion induces monopolar spindles [58], [59]. The latter monopolarity, however, can be suppressed by the loss of Ndc80 mediated kinetochore-microtubule attachments, achieved by co-depletion of both CENP-O and Nuf2R proteins [59]. Fine regulation of microtubule properties by CCAN components has been reported, with the CENP-H/I complex regulating microtubule dynamics and metaphase chromosome oscillations, much like a clutch engaging the engine of microtuble flux [60]. As a component of the CENP-T/W/S/X particle, it is likely that CENP-W depletion is working primarily through the Ndc80 pathway, but the behavior of spindles indicates that force distribution regulated by non-kinetochore motor pathways is affected as well. The kinetochore serves as a platform for the binding and assembly of protein complexes as well as an attachment site for microtubules. Our work suggests that the CCAN and CENP-W in particular may play a functional role in this, coordinating the scaffolding function of the kinetochore with the dynamic chromatin-microtubule interface.

## Supporting Information

File S1 **File contains Figures S1–S6.** Figure S1. Cohesion fatigue is unlikely to be the main mechanism driving multipolarity in CENP-W depleted cells. (A) Representative panel of HeLa H2B-GFP cells depleted of CENP-W for 48 h and stained with ACA (blue) and Zwint-1 (red) for centromere and kinetochore localization, respectively. The vast number of scattered chromosomes contain clearly visible coupled centromeres (box 2) while some display split sister kinetochores (box 1). (B) Representative panel of GFP-CENP-W HeLa cells depleted of CENP-W for 48 h and stained for pericentrin (green) and centrin-3 (red), red arrows indicate the appearance of non-centriolar pole, which can originate as a consequence of spindle pole defects associated with centriole disengagement (cell #1) or mechanical disruption of the pole with normal paired centrioles (cell #2). Figure S2. Depletion of CENP-W protein in GFP-CENP-W HeLa cells. (A) HeLa cells expressing GFP-CENP-W were transfected with pooled siRNA for CENP-W (CW), CENP-T (CT) and non-targeting (NT) control. Samples harvested at indicated times were Western blotted against GFP and CENP-W for detection of a tagged and endogenous protein, respectively. Numbers represent relative protein abundance, measured by quantification of chemiluminescence signal and normalized to tubulin. (B) Representative image of GFP-CENP-W (green) cells, depleted for CENP-W and stained with DAPI (blue), upper panel. Quantitative measurements of the mean (+SEM) GFP fluorescence signal intensities of the mitotic prometaphases and metaphases cells, following siRNA transfection, lower panel. Values plotted were background corrected. *** p<0.0001 (one-way ANOVA followed by Dunnetts multiple comparison). Figure S3. Mad1/Mad2 complex is depleted from the kinetochores following siCENP-W. Representative panels of HeLa H2B-GFP cells treated with 1 µM nocodazole for 12 h (for maximized mitotic checkpoint protein induction) or cells depleted of CENP-W with siRNA for 48 h. GFP channel was used to visualize chromosomes of cells stained for Zwint-1 (green) and different checkpoint proteins detection (red): (A) BubR1, (B) Mad1 and (C) Mad2. In the overlay, blue signal corresponds to ACA staining for centromers visualization and yellow indicates co-localization of the two outer kinetochore proteins. The boxed enlargements show co-localization (or lack) of the Mad1 with outer kinetochore marker (B) or accumulation of Mad2 checkpoint protein at the spindle poles (C) Scale bar 5 µm. (A, B, C) Graphs represent mean percentage (+SEM) of checkpoint positive kinetochores exhibiting strong fluorescence intensities (at least 1000 kinetochores were scored, in duplicate assays). *** p<0.0001 (one-way ANOVA followed by Dunnetts multiple comparison). Figure S4. Suppression of supernumerary γ-tubulin foci in nocodazole siCENP-W treated cells. Representative panel of GFP-CENP-W cells transfected with siCENP-W for 48 h, incubated with 1 µM nocodazole (NOC) for the last 4 h of transfection and stained for γ-tubulin (green) and α-tubulin (red). Scale bar 10 µm. Figure S5. Evaluation of the overexpression of hTPX2 in GFP-CENP-W HeLa cells over time. (A) Representative panel of GFP-CENP-W HeLa cells overexpressing YFP-TPX2 protein, showing similar to the endogenous localization pattern. Scale bar 5 µm. (B) HeLa cells expressing GFP-CENP-W were transiently transfected with YFP-TPX2 and/or non-targeting (NT) control siRNA. Samples harvested at indicated times were Western blotted and probed against TPX2 for detection of a tagged and endogenous protein. Tubulin was used as the protein loading control. (C) Representative image of GFP-CENP-W cells, fixed at indicated times, overexpressing YFP-TPX2 protein (green) and stained with DAPI (blue). Figure S6. Localization of the endogenous hTPX2 in HeLa cells. (A) Exponentially growing HeLa cells were fixed with 4% PFA and processed for immunofluorescence with antibody against hTPX2 (green) and α-tubulin (red). Co-localization across the different stages of mitosis of the spindles with hTPX2 is shown in yellow, in the overlay. Scale bar 5 µm.
(PDF)
