# Supplementary material for: CENP-W Plays a Role in Maintaining Bipolar Spindle Structure
Source: PLoS One. 2014 Oct 15;9(10):e106464. doi: 10.1371/journal.pone.0106464 (PMC4198083; doi:10.1371/journal.pone.0106464)
Supplement: File S1 — File contains Figures S1–S6. Figure S1. Cohesion fatigue is unlikely to be the main mechanism driving multipolarity in CENP-W depleted cells. (A) Representative panel of HeLa H2B-GFP cells depleted of CENP-W for 48 h and stained with ACA (blue) and Zwint-1 (red) for centromere and kinetochore localization, respectively. The vast number of scattered chromosomes contain clearly visible coupled centromeres (box 2) while some display split sister kinetochores (box 1). (B) Representative panel of GFP-CENP-W HeLa cells depleted of CENP-W for 48 h and stained for pericentrin (green) and centrin-3 (red), red arrows indicate the appearance of non-centriolar pole, which can originate as a consequence of spindle pole defects associated with centriole disengagement (cell #1) or mechanical disruption of the pole with normal paired centrioles (cell #2). Figure S2. Depletion of CENP-W protein in GFP-CENP-W HeLa cells. (A) HeLa cells expressing GFP-CENP-W were transfected with pooled siRNA for CENP-W (CW), CENP-T (CT) and non-targeting (NT) control. Samples harvested at indicated times were Western blotted against GFP and CENP-W for detection of a tagged and endogenous protein, respectively. Numbers represent relative protein abundance, measured by quantification of chemiluminescence signal and normalized to tubulin. (B) Representative image of GFP-CENP-W (green) cells, depleted for CENP-W and stained with DAPI (blue), upper panel. Quantitative measurements of the mean (+SEM) GFP fluorescence signal intensities of the mitotic prometaphases and metaphases cells, following siRNA transfection, lower panel. Values plotted were background corrected. *** p<0.0001 (one-way ANOVA followed by Dunnetts multiple comparison). Figure S3. Mad1/Mad2 complex is depleted from the kinetochores following siCENP-W. Representative panels of HeLa H2B-GFP cells treated with 1 µM nocodazole for 12 h (for maximized mitotic checkpoint protein induction) or cells depleted of CENP-W with siRNA for 48 h. GFP c [file pone.0106464.s001.pdf]

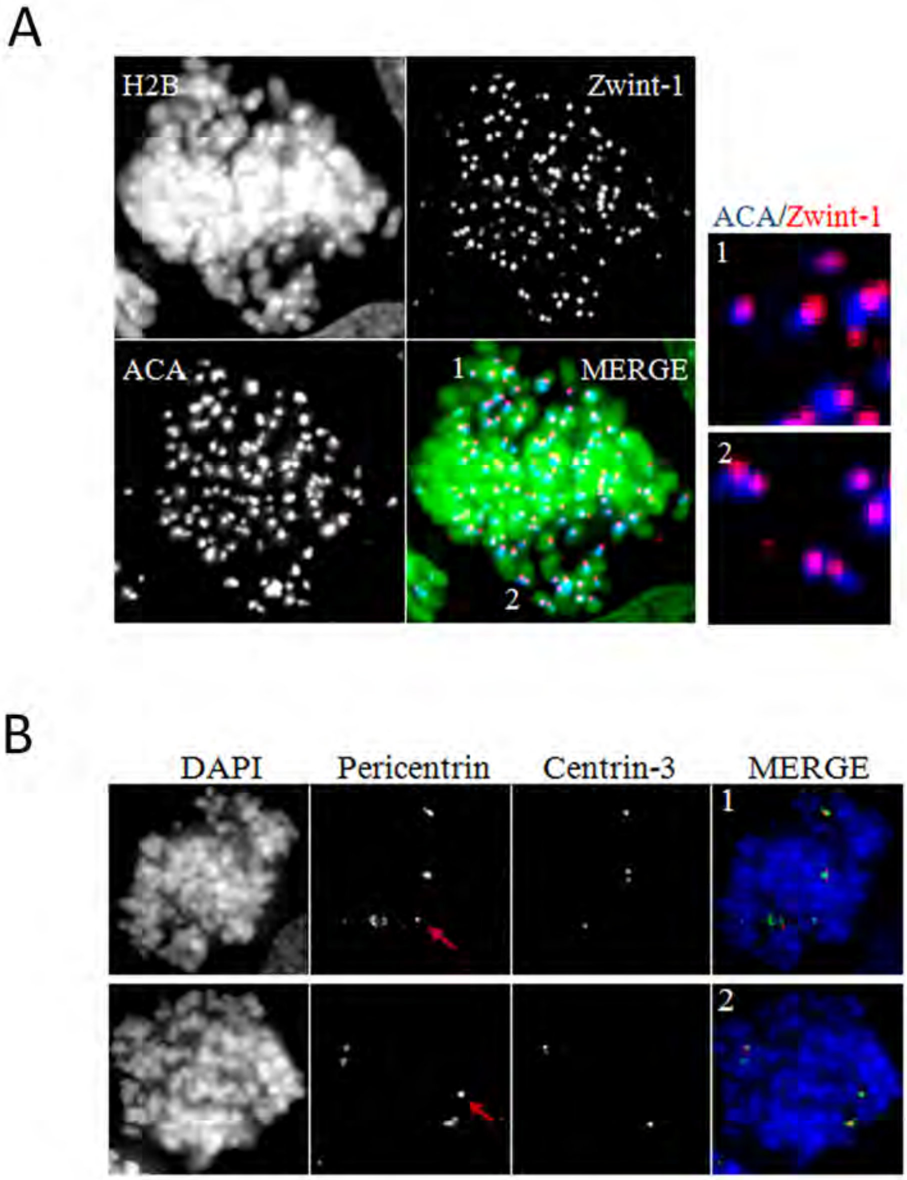

Supplementary Figure S1. Cohesion fatigue is unlikely to be the main mechanism driving multipolarity in CENP-W depleted cells. (A) Representative panel of HeLa H2B-GFP cells depleted of CENP-W for 48h and stained with ACA (blue) and Zwint-1 (red) for centromere and kinetochore localization, respectively. The vast number of scattered chromosomes contain clearly visible coupled centromeres (box 2) while some display split sister kinetochores (box 1).
(B) Representative panel of GFP-CENP-W HeLa cells depleted of CENP-W for 48h and stained for pericentrin (green) and centrin-3 (red), red arrows indicate the appearance of non-centriolar pole, which can originate as a consequence of spindle pole defects associated with centriole disengagement (cell #1) or mechanical disruption of the pole with normal paired centrioles (cell #2).

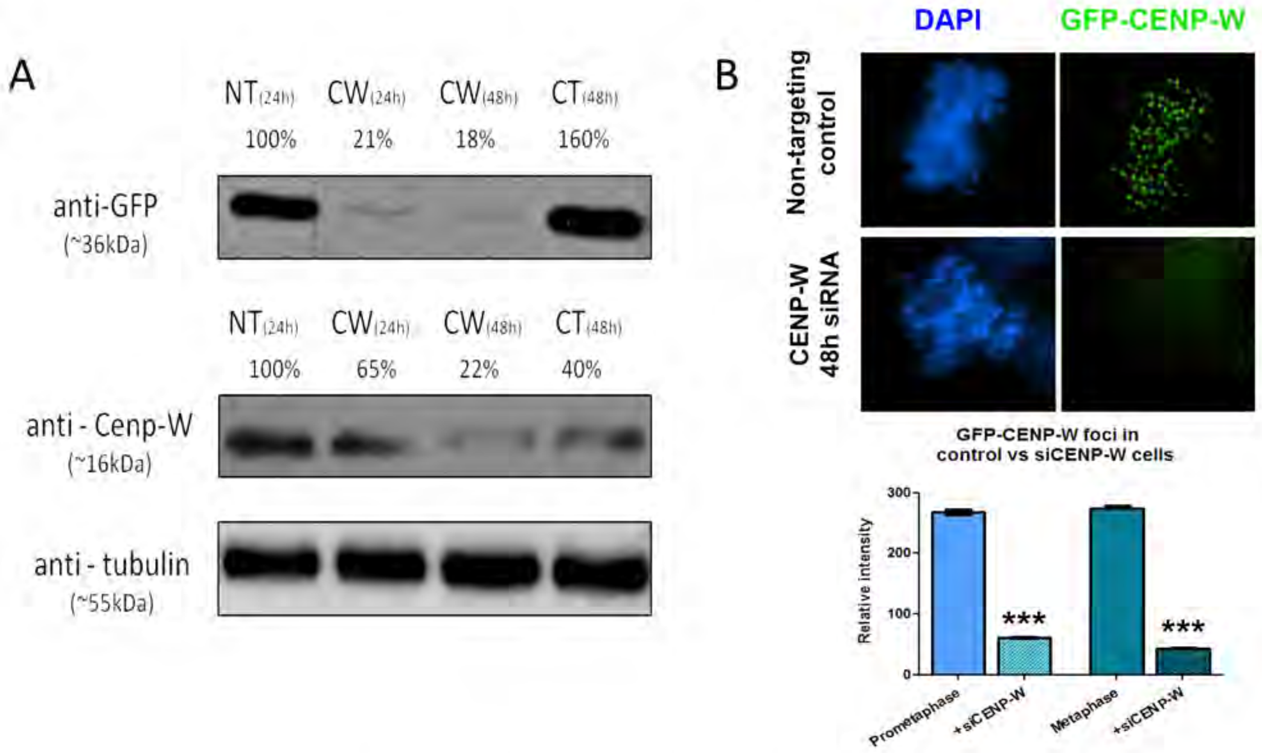

Supplementary Figure S2. Depletion of CENP-W protein in GFP-CENP-W HeLa cells. (A) HeLa cells expressing GFP-CENP-W were transfected with pooled siRNA for CENP-W (CW), CENP-T (CT) and non-targeting (NT) control. Samples harvested at indicated times were Western blotted against GFP and CENP-W for detection of a tagged and endogenous protein, respectively. Numbers represent relative protein abundance, measured by quantification of chemiluminescence signal and normalized to tubulin. (B) Representative image of GFP-CENP-W (green) cells, depleted for CENP-W and stained with DAPI (blue), upper panel. Quantitative measurements of the mean (+SEM) GFP fluorescence signal intensities of the mitotic prometaphases and metaphases cells, following siRNA transfection, lower panel. Values plotted were background corrected. *** $p<0.0001$ (one-way ANOVA followed by Dunnetts multiple comparison).

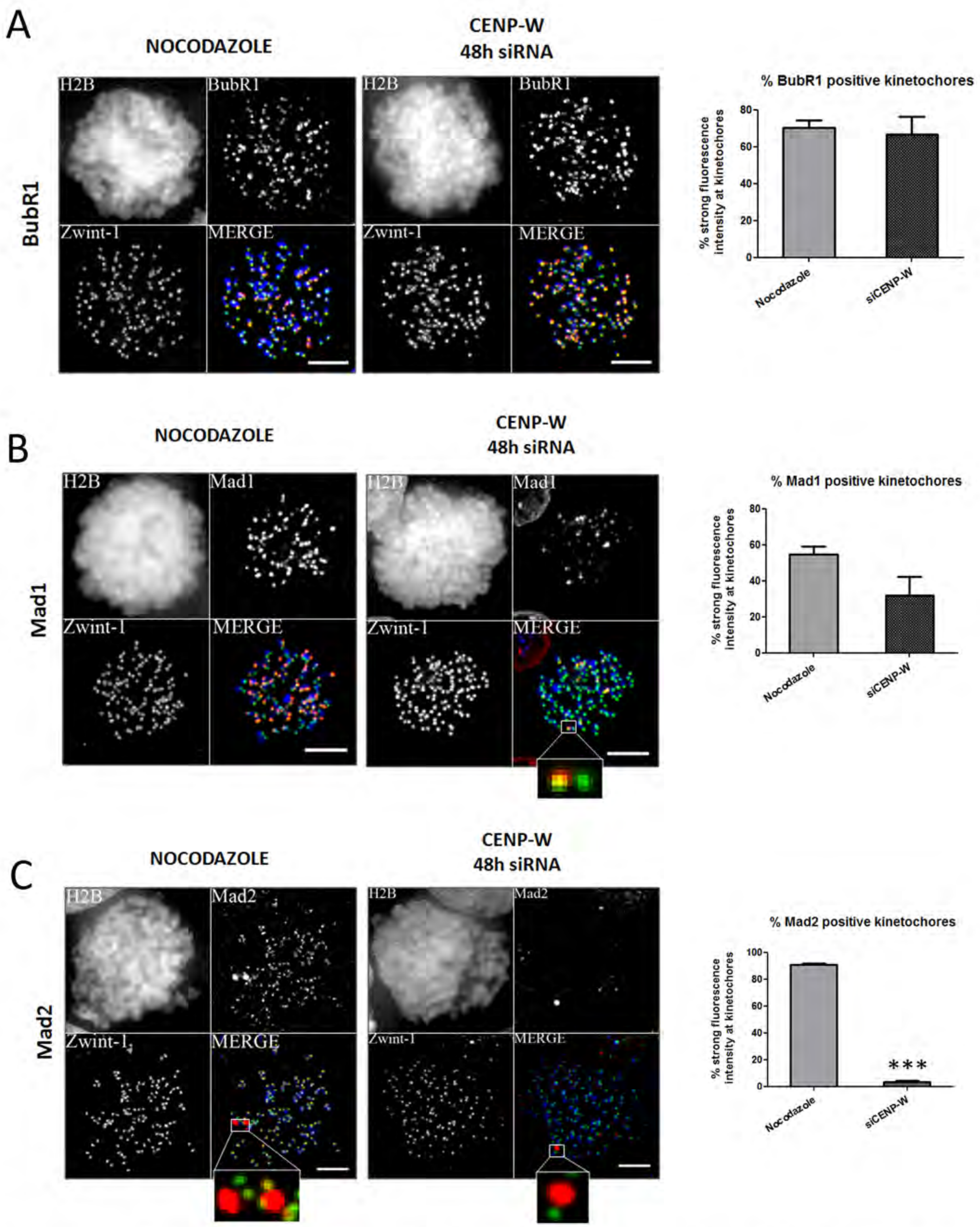

Supplementary Figure S3. Mad1/Mad2 complex is depleted from the kinetochores following siCENP-W.
Representative panels of HeLa H2B-GFP cells treated with 1µM nocodazole for 12h (for maximized mitotic checkpoint protein induction) or cells depleted of CENP-W with siRNA for 48h. GFP channel was used to visualize chromosomes of cells stained for Zwint-1 (green) and different checkpoint proteins detection (red): (A) BubR1, (B) Mad1 and (C) Mad2. In the overlay, blue signal corresponds to ACA staining for centromers visualization and yellow indicates co-localization of the two outer kinetochore proteins. The boxed enlargements show co-localization (or lack) of the Mad1 with outer kinetochore marker (B) or accumulation of Mad2 checkpoint protein at the spindle poles (C) Scale bar 5µm.
(A, B, C) Graphs represent mean percentage (+SEM) of checkpoint positive kinetochores exhibiting strong fluorescence intensities (at least 1000 kinetochores were scored, in duplicate assays). *** $p<0.0001$ (one-way ANOVA followed by Dunnetts multiple comparison).

A

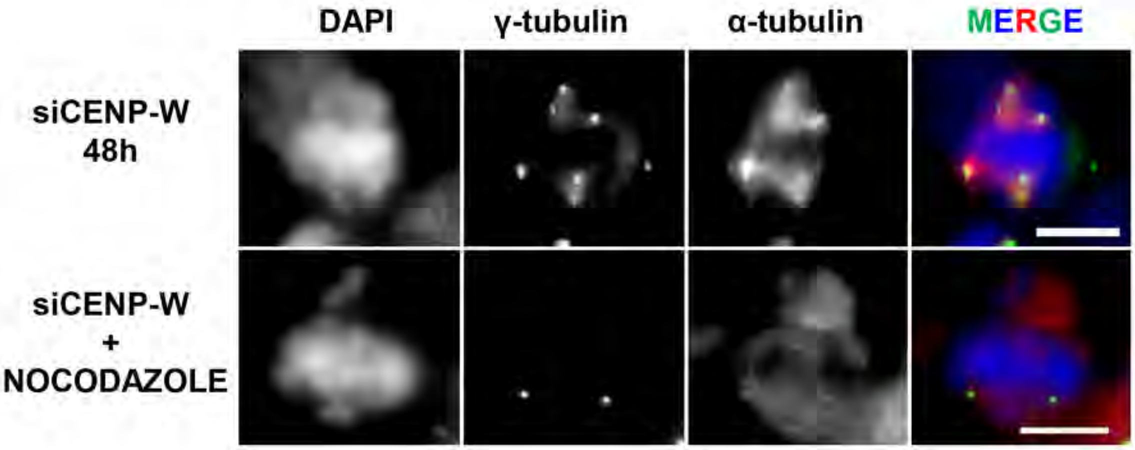

Supplementary Figure S4. Suppression of supernumerary γ-tubulin foci in nocodazole siCENP-W treated cells. Representative panel of GFP-CENP-W cells transfected with siCENP-W for 48h, incubated with 1μM nocodazole (NOC) for the last 4h of transfection and stained for γ-tubulin (green) and α-tubulin (red). Scale bar 10μm.

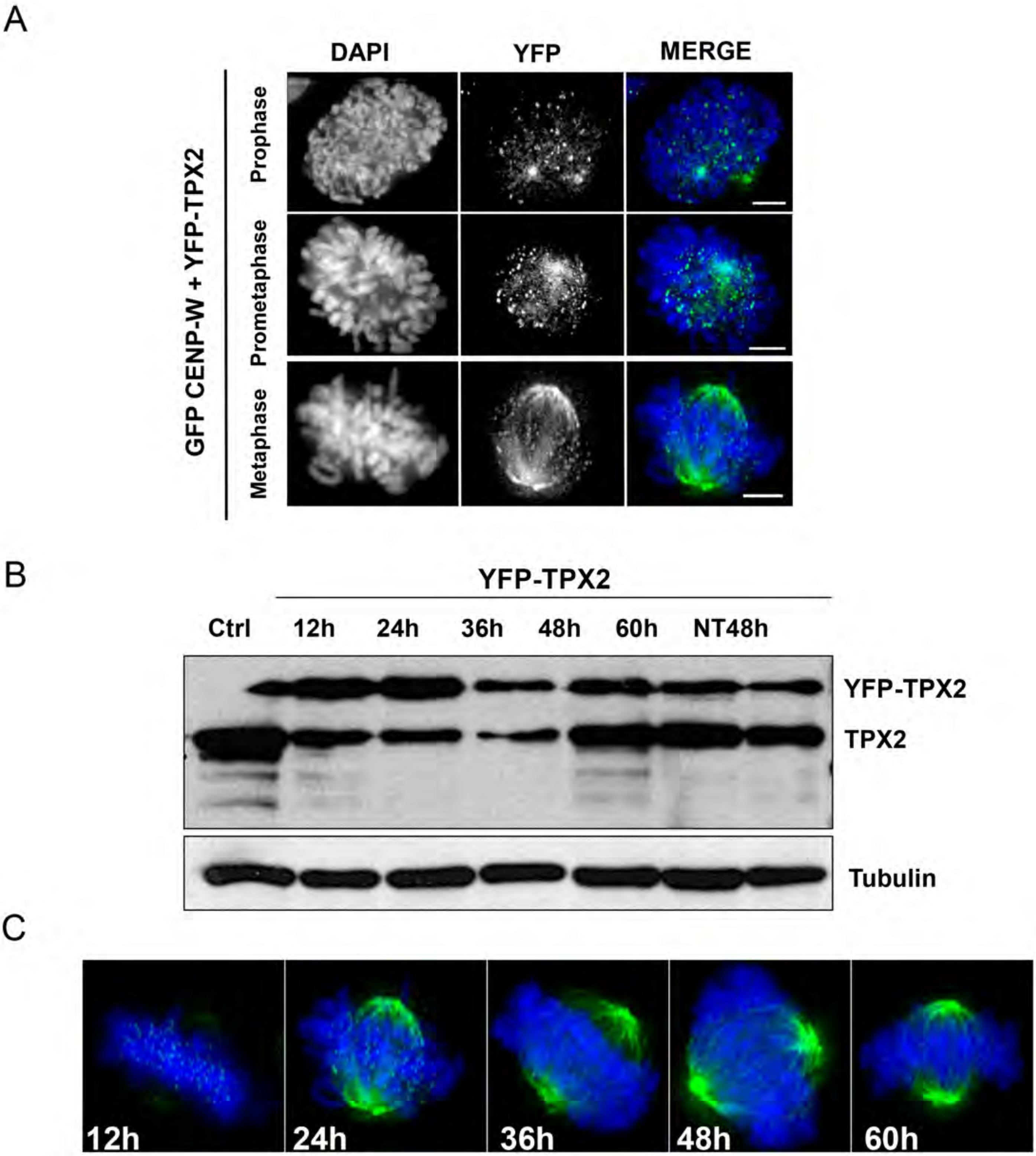

Supplemenary Figure S5. Evaluation of the overexpression of hTPX2 in GFP-CENP-W HeLa cells over time. (A) Representative panel of GFP-CENP-W HeLa cells overexpressing YFP-TPX2 protein, showing similar to the endogenous localization pattern. Scale bar 5μm. (B) HeLa cells expressing GFP-CENP-W were transiently transfected with YFP-TPX2 and/or non-targeting (NT) control siRNA. Samples harvested at indicated times were Western blotted and probed against TPX2 for detection of a tagged and endogenous protein. Tubulin was used as the protein loading control. (C) Representative image of GFP-CENP-W cells, fixed at indicated times, overexpressing YFP-TPX2 protein (green) and stained with DAPI (blue).

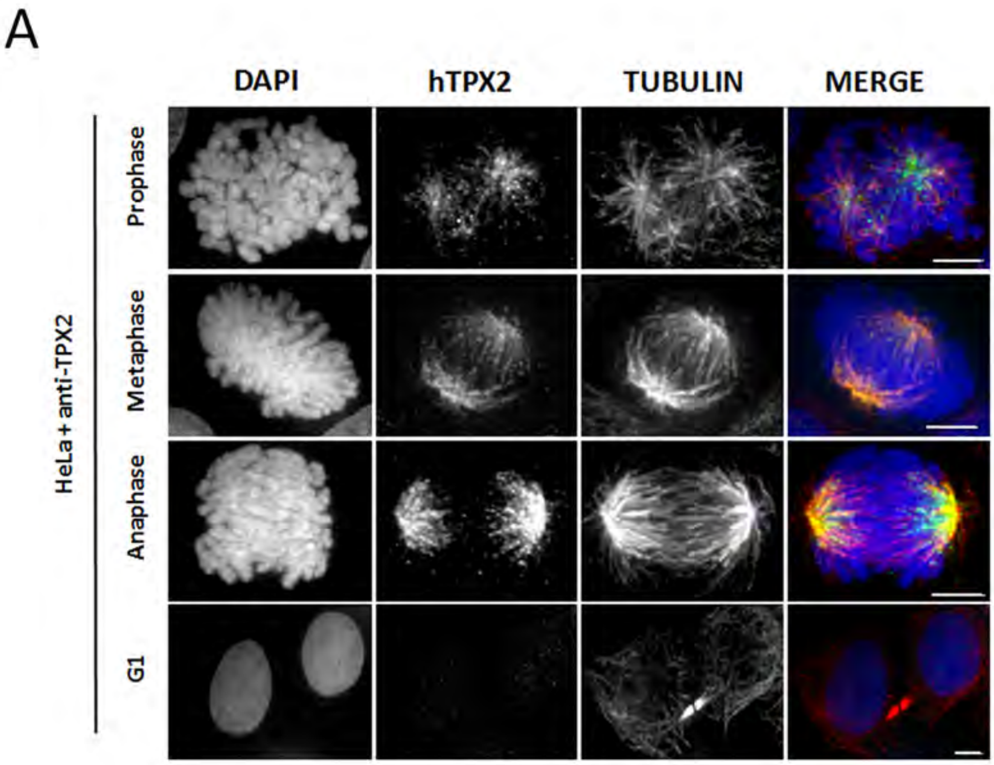

Supplementary Figure S6. Localization of the endogenous hTPX2 in HeLa cells. (A) Exponentially growing HeLa cells were fixed with 4% PFA and processed for immunofluorescence with antibody against hTPX2 (green) and α-tubulin (red). Co-localization across the different stages of mitosis of the spindles with hTPX2 is shown in yellow, in the overlay. Scale bar 5μm.
